# Alkaloid Profile of Fifteen Different Species of *Narcissus* L. (Amaryllidoideae) Collected in Spain

**DOI:** 10.3390/plants14172793

**Published:** 2025-09-06

**Authors:** María Lenny Rodríguez-Escobar, Vanessa Martínez-Francés, Segundo Ríos, Gabriela E. Feresin, Warley de Souza Borges, Jaume Bastida, Laura Torras-Claveria, Luciana R. Tallini

**Affiliations:** 1 Departament de Biologia, Sanitat i Medi Ambient, Facultat de Farmàcia i Ciències de l’Alimentació, Universitat de Barcelona, Av. Joan XXIII 27-31, 08028 Barcelona, Spain; mrodries116@alumnes.ub.edu (M.L.R.-E.); jaumebastida@ub.edu (J.B.); 2 Departamento de Biología Aplicada, Area de Botánica, Universidad Miguel Hernández, Av. Universidad, s/n, 03202 Elche, Spain; vanessa.martinezf@umh.es; 3 Estación Biológica Torretes-Jardín Botánico de la UA, Universidad de Alicante, Crtra. Sant Vicent del Raspeig, s/n, 03690 Alicante, Spain; s.rios@ua.es; 4 Instituto de Biotecnología, Facultad de Ingeniería, Universidad Nacional de San Juan, Av. Libertador General San Martin 1109 Oeste, San Juan 5400, Argentina; gferesin@unsj.edu.ar; 5 Consejo Nacional de Investigaciones Científicas y Técnicas (CONICET), CCT CONICET, San Juan 5400, Argentina; 6 Laboratório de Produtos Naturais, Departamento de Química, Universidade Federal do Espírito Santo, Vitoria 29075-910, Brazil; warley.borges@ufes.br

**Keywords:** Amaryllidoideae, daffodils, gas chromatography, molecular biodiversity, nitrogenate metabolites

## Abstract

Molecular diversity is a key component of overall biodiversity, playing a vital role in evolution. It results from the adaptation of organisms to various habitats, which impacts their survival. The Amaryllidoideae subfamily is a significant group of monocotyledonous plants known for producing an exclusive and still-expanding group of molecules with diverse biological activities. Galanthamine (Gal), the most renowned metabolite from Amaryllidoideae subfamily, has been marketed for the palliative treatment of Alzheimer’s disease since 2001 due to its ability to inhibit the acetylcholinesterase enzyme. Due to the high cost and low yield of its synthesis, pharmaceutical companies extract this drug from Amaryllidoideae plants, such as *Narcissus pseudonarcissus* cv. Carlton in Europe and *Lycoris radiata* in China. The aim of this study was to describe the alkaloid profile of fifteen different species of *Narcissus* L. (commonly known as daffodils) collected in Spain using gas chromatography coupled with mass spectrometry. Fifty-one alkaloids were identified and quantified within these species through our private library of Amaryllidaceae alkaloids (AA) built over the last four decades, while thirty structures remained not identified in thirteen of these species. The highest concentration of these nitrogenate metabolites was quantified in *N. confusus*, 541 μg Gal·100 mg^−1^ DW, which also exhibited a notably high concentration of Gal, 301 μg Gal·100 mg^−1^ DW, which represents about 55% of the alkaloids identified in this species. The species *N. bujei* was also found to contain a significant quantity of this compound, amounting to 103.2 μg Gal·100 mg^−1^ DW. The plant *N. assoanus* harbored a total of seven unidentified compounds, indicating that this species could be a potentially important source of novel alkaloids. In conclusion, this study facilitates a direct comparison of alkaloid profiles for fifteen *Narcissus* plant species. This serves as a valuable tool for identifying possible new sources of galanthamine, as well as other novel medicinal alkaloids. Finally, this work presents the first alkaloid profile of the species *N. minor* and *N. nevadensis*.

## 1. Introduction

Plants constitute an important pillar of terrestrial ecosystems, providing a vast array of bioresources essential for human nutrition, healthcare, cultural practices, and technological advancements [1]. Plants from the subfamily Amaryllidoideae (Amaryllidaceae) are widely appreciated and cultivated for their ornamental value. This subfamily includes more than 60 genera distributed worldwide, with *Narcissus* L., commonly known as daffodils, being the most well-known in Europe. This genus, which comprises around 100 species, has a significant center of diversity in the Iberian Peninsula, particularly in Spain [2].

Amaryllidoideae plants also have medicinal value owing to the biological activity of the alkaloids they can biosynthesize. These compounds, known as Amaryllidaceae alkaloids (AA), are isoquinoline-type alkaloids, and they constitute a distinctive chemotaxonomic feature of this subfamily [3]. These molecules have demonstrated extensive biological activities, such as antiviral, antifungal, antibacterial, antimalarial, insecticide, cytotoxic, antitumor, and antifungal activities, as well as the inhibition of acetylcholinesterase (AChE), among others [4,5,6,7].

Over 600 AA have been reported to date, all originating from the precursor norbelladine, which is formed from L-phenylalanine and L-tyrosine. This precursor diverged into different skeleton types through three phenol-oxidative couplings: *o*-*p*’ (leading to lycorine and homolycorine types), *p*-*p*’ (leading to haemanthamine, crinine, narciclassine, montanine, and tazettine types), and *p*-*o*’ (leading to galanthamine-type alkaloids). The final diversity of these alkaloids is then achieved through reactions like reduction, oxidation, hydroxylation, methylation, condensation, and oxide bridge formation [3,8].

Galanthamine, an acetylcholinesterase (AChE) inhibitor, is an Amaryllidaceae alkaloid that has emerged as a valuable therapeutic agent in the palliative management of Alzheimer’s disease (AD) symptoms following its FDA approval in 2001. This drug is able to traverse the blood–brain barrier, and its long-lasting, selective, reversible, and competitive inhibitory properties make it a valuable pharmacological tool in combating AD-associated cognitive decline. This medication is commercialized under the brand names Razadine^®^ in the USA and Reminyl^®^ in Europe [9,10]. Moreover, following patent expiration, it has become available as a generic drug, expanding its accessibility and affordability for AD patients. This alkaloid is obtained from natural sources within the Amaryllidoideae subfamily by pharmaceutical companies. They extract it from plants such as *Narcissus pseudonarcissus* L. cv. Carlton in Europe and *Lycoris radiata* (L’Hérit.) Herb in China. However, several problems, including unsuccessful cultivation or slow regeneration, make it difficult to meet increasing pharmaceutical demands. Gal can also be produced by chemical synthesis, but the yield of the total synthesis is still considered too low to be economically feasible [11].

In a recent publication, we described the acetylcholinesterase (AChE)- and butyrylcholinesterase (BuChE)-inhibitory potential of fifteen different *Narcissus* L. species collected in Spain [12]. In this way, the aim of this current work was to provide the alkaloid profile of these same fifteen species of *Narcissus* using gas chromatography coupled to mass spectrometry (GC-MS).

## 2. Results and Discussion

### 2.1. Identified Alkaloids

**Figure 1:**
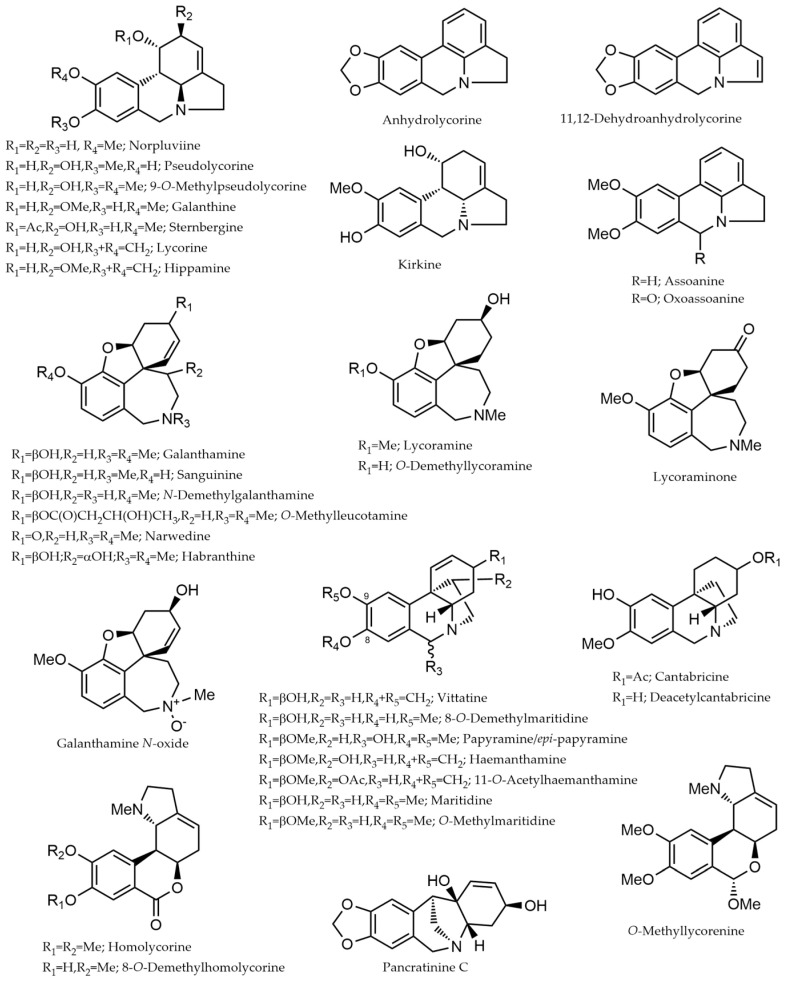

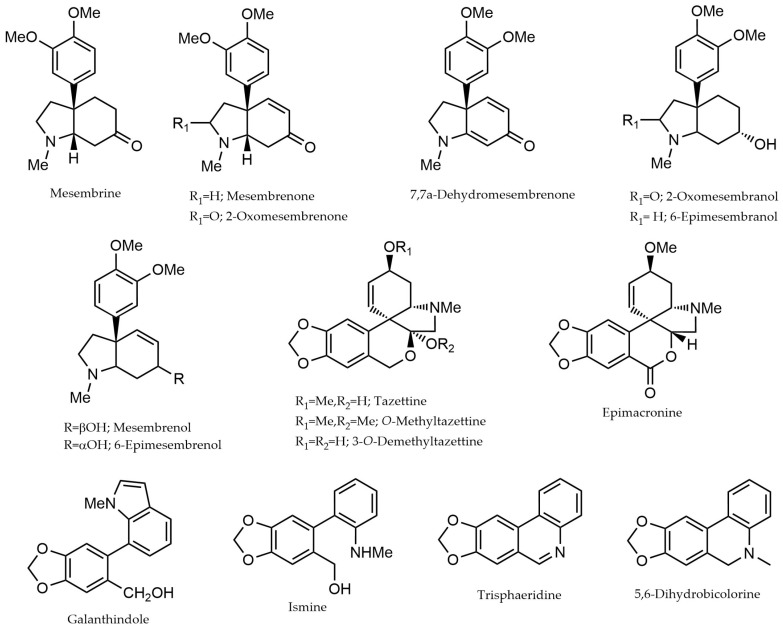
Chemical structure of the alkaloids identified in the different species of *Narcissus* L. by GC-MS. The structures were drawn using ChemDraw (version 2023).

Fifty-one alkaloids have been identified and quantified in the leaf extract of fifteen different species of *Narcissus* (Table 1). Due to limitations in obtaining reference compounds for the absolute quantification of each alkaloid determined in these samples, Gal was used for this purpose. Therefore, the values described in Table 1 represent the alkaloid content expressed as micrograms of Gal equivalents per 100 milligrams of dry weight (DW) of plant material (μg Gal·100 mg^−1^ DW). The chemical structures of these alkaloids are represented in Figure 1.

Table 1 facilitates the comparison of the alkaloid profiles across the fifteen *Narcissus* species analyzed herein. Detailed information regarding the yield of each extract is presented by Tallini and co-authors [12]. According to this, *N. yepesii* exhibited the highest yield (6.14%), significantly exceeding the average yield of 1.32% observed among the remaining species [12].

Evaluating the results expressed in Table 1, the highest quantity of identified alkaloids was observed in the extract of *N. confusus* (sample E), followed by the species *N. bujei* (sample I), with a total of 541.0 and 523.2 μg Gal·100 mg^−1^ DW, respectively. On the other hand, the species *N. vasconicus* (sample C) and *N. minor* (sample D) presented the lowest concentration of identified alkaloids, with values of 63.2 and 67.1 μg Gal·100 mg^−1^ DW, respectively (Table 1).

The species *N. confusus* stands out among the species tested for showing high quantities of galanthamine-type alkaloids, specially galanthamine, with 301.0 μg Gal·100 mg^−1^ DW, which represents 55.6% of its total identified alkaloids. This translates to a yield of 0.3% (of DW) of galanthamine from the leaves of this species. The high concentration of galanthamine in this sample is consistent with previously documented levels reported in the literature [13]. As shown in Table 1, alongside galanthamine-type structures, *N. confusus* also presented alkaloids from haemanthamine-, pretazettine-, homolycorine-, narciclasine-, ismine-, and galanthindole-type alkaloids. In Europe, the bulbs of the species *Narcissus pseudonarcissus* cv. Carlton are used by pharmaceutical companies to obtain galanthamine, which contains about 0.12–0.15% (of DW) of this drug and significant quantities of haemanthamine and narciclasine [11].

Among all the species evaluated herein, *N. bujei* (sample I) was the second most representative in galanthamine. This species showed 103.2 μg Gal·100 mg^−1^ DW of this product, which represents about 20% of the alkaloids content identified in this sample. The presence of structures belonging to other scaffolds have also been observed in this species, represented by high concentrations of the alkaloids haemanthamine and homolycorine, with values of 141.1 and 135.8 μg Gal·100 mg^−1^ DW, respectively (Table 1).

The sample *N. pallidulus* (sample J) showed a high diversity of alkaloids from the *Sceletium*-type group, which are also known as mesembrine-type alkaloids. Six structures with this alkaloid-type skeleton have been identified in this species, totaling 139.9 μg Gal·100 mg^−1^ DW. Among them, the alkaloid mesembrenone was the most representative, with 71.0 μg Gal·100 mg^−1^ DW (see Table 1). Previous works also report the presence of different structures from *Sceletium*-type alkaloids in *N. pallidulus* [14,15]. This scaffold is the only group of structures that is not exclusively of the monocotiledon subfamily Amaryllidoideae, being typical alkaloids of the genus *Sceletium*, which belongs to the dicotyledonous family Aizoaceae, with a center of origin in South Africa [14,16]. According to Table 1, three alkaloids from the haemanthamine, pretazettine, and homolycorine groups have also been detected in the leaf extracts of *N. pallidulus*, but in a very low concentration, totaling 14.6 μg Gal·100 mg^−1^ DW.

In addition to *N. pallidulus*, the presence of *Sceletium*-type alkaloids was also observed in the species *N. minor* (sample D), specifically mesembrenone and 2-oxomesembrenone, with 2.8 and 3.5 μg Gal·100 mg^−1^ DW, respectively. This species also showed alkaloids belonging to galanthamine-, homolycorine-, and lycorine-type scaffolds (Table 1). Along with *N. nevadensis* (sample O), this is the first report about the alkaloid profiling of these two plant species. In *N. nevadensis*, it was possible to identify lycorine- and homolycorine-type alkaloids, with homolycorine being the most abundant compound at 34.7 μg Gal·100 mg^−1^ DW (Table 1).

The species *N. confusus* (sample E) and *N. asturiensis* (sample F) presented the highest amount of pretazettine-type alkaloids, of which tazettine was the major representative, with 53.2 and 58.7 μg Gal·100 mg^−1^ DW, respectively (Table 1). This alkaloid is an artifact of pretazettine, which shows no EI mass fragmentation under GC–MS conditions due to its rapid conversion to tazettine in the chromatographic column [17].

Among all the species reported in Table 1, only *N. yepesii* (sample N) showed the presence of montanine-type alkaloids, represented by pancratinine C (25.1 μg Gal·100 mg^−1^ DW), which represents around 10% of its total content of identified alkaloids (260.8 μg Gal·100 mg^−1^ DW). This alkaloid scaffold comprises a limited group of fourteen structures isolated from Amaryllidaceae genera that lack phylogenetic relatedness and is very unusual among *Narcissus* species [3].

### 2.2. Not Identified Alkaloids

All the alkaloids quantified in the leaf extracts of the species *N. bujei* (sample I) and *N. yepesii* (sample O) have been identified (Table 1). However, it was not possible to identify a total of thirty alkaloids distributed among the other thirteen species evaluated herein (Table 2).

*N. assoanus* (sample A) harbored the most diverse range of not identified structures, with a total of seven, followed by *N. hedraeanthus* (sample G), with five not identified alkaloids. According to Table 2, two species, *N. jacetanus* (sample B) and *N. pallidulus* (sample J), exhibited four not identified structures each, while one species, *N. minor* (sample D), contained three not identified structures. Most of the remaining species had zero-to-two not identified alkaloids in their chemical composition (Table 2).

In addition to showing the greatest diversity of not identified alkaloids, the species *N. assoanus* also presented the highest quantity of these structures, totaling 76.4 μg Gal·100 mg^−1^ DW. The second sample with the highest content of not identified alkaloids was *N. confusus* (sampe E), containing 61.8 μg Gal·100 mg^−1^ DW of the compound NI-25. Alongside *N. confusus*, *N. alcaracensis* (sample H) and *N. genesii-lopezii* (sample M) also presented high amounts of this structure, which could be a new compound. Consistent with previous reports [18], the presence of a characteristic base peak at *m*/*z* 109 in the fragmentation pattern of NI-25 (Table 2) suggests that this molecule belongs to the homolycorine-type skeleton and shows a double bond among C-3 and C-4. The species *N. minor* and *N. nevadensis*, whose alkaloid profiles are described here for the first time, each exhibited two not identified alkaloids.

According to the literature, an investigation into the alkaloid profile of 40 ornamental *Narcissus* taxa was conducted to identify suitable sources of biologically active compounds, such as Gal, lycorine, and haemanthamine, for commercial production [19]. Among all the samples, the authors detected 97 typical AA, of which 49 were not identified [19].

### 2.3. Alkaloid Type Distribution Among the Species

**Figure 2:**
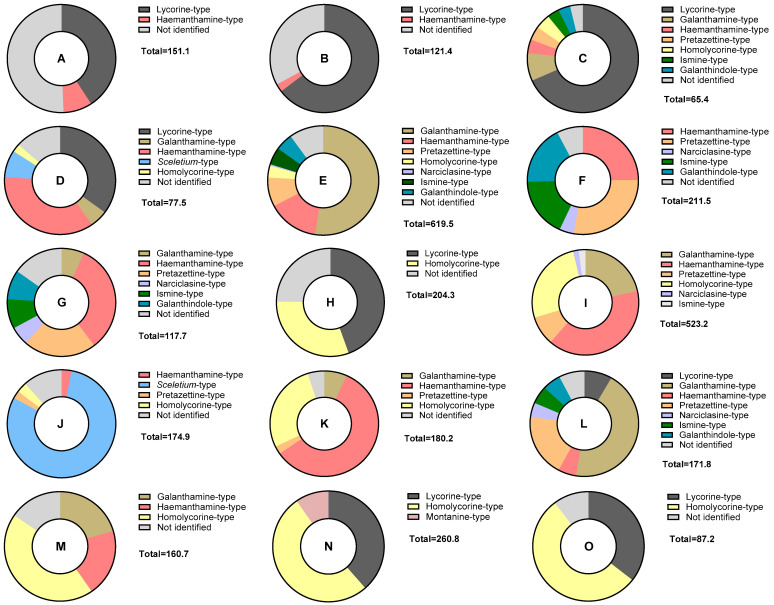
Doughnut chart representing the percentage of identified alkaloid-type scaffolds and not identified structures quantified in each *Narcissus* L. extract using GC-MS analysis. Total values are in μg Gal·100 mg^−1^ DW. A = *N. assoanus*; B = *N. jacetanus*; C = *N. vasconicus*; D = *N. minor*; E = *N. confusus*; F = *N. asturiensis*; G = *N. hedraeanthus*; H = *N. alcaracensis*; I = *N. bujei*; J = *N. pallidulus*; K = *N. tazetta*; L = *N. jonquilla*; M = *N. genesii-lopezii*; N = *N. yepesii*; O = *N. nevadensis*.

To represent the distribution of different alkaloid types and not identified alkaloids in each *Narcissus* L. extract, a doughnut chart was generated for every sample. These charts represent the percentage of identified alkaloid-type scaffolds and not identified structures quantified in the fifteen different species of *Narcissus* using GC-MS analysis (Figure 2). This type of graphical representation provides a comprehensive overview of each sample. Depicting the relative abundance of identified and not identified alkaloids provides a deeper understanding of the overall alkaloid profile for each species and helps identify species that could be a source of novel bioactive alkaloids.

In the doughnut chart in Figure 2, it can be observed that the not identified alkaloids (in light gray color) represent an important percentage in the samples A, B, and H (*N. assoanus*, *N. jacetanus,* and *N. alcaracensis*, respectively). On the other hand, this analysis highlights the significant potential of samples E, I, L, and M (*N. confusus*, *N. bujei*, *N. jonquilla*, and *N. genesii-lopezii*) as a source of galanthamine-type structures, represented by the mustard yellow color.

Samples A and L belong to the section Jonquillae; samples B, C, D, E, F, I, and M to Pseudonarcissus; H, N, and O to Nevadensis; and J, K, and G to Ganymedes, Tazettae, and Bulbocodium, respectively [12]. Taking the sections into account, the species belonging to section Jonquillae, *N. assoanus* (A) and *N. jonquilla* (L), did not show high similarity in their alkaloid profiles. In contrast, all species from the Pseudonarcissus section exhibited alkaloids of the haemanthamine type. The species *N. nevadensis* (O), after which the section Nevadensis was named, contains alkaloids of the lycorine and homolycorine types, similar to other species in the same section, such as *N. alcaracensis* (H) and *N. yepesii* (N) (Figure 2).

Based on a comprehensive review of the available literature, our previous publication [12] provided a detailed overview of the alkaloid previously identified in these same fifteen species of *Narcissus* L. Synthesizing the information previously published, the following structures belonging to the alkaloid-type skeletons, namely, bujeine, ismine, galanthamine, haemanthamine, homolycorine, lycorine, montanine, narciclasine, norbelladine, pretazettine, and *Sceletium*, have previously been reported among these species [13,15,16,20,21,22,23,24,25,26,27,28,29,30,31,32,33,34]. Detailed information is available in Table 3. No prior information regarding the alkaloid content of the species *N. minor* (sample D) and *N. nevadensis* (sample O) has been found in the literature.

On the other hand, in a recent publication [12], we described the acetylcholinesterase (AChE)- and butyrylcholinesterase (BuChE)-inhibitory potential of the same species of *Narcissus* reported herein. According to our previous results [12], *N. jacetanus* exhibited the most potent AChE inhibition, with an IC_50_ value of 0.75 ± 0.03 μg/mL, while *N. jonquilla* demonstrated the strongest BuChE inhibition, with an IC_50_ value of 11.72 ± 1.15 μg/mL [12]. Comprehensive alkaloid profiling of *N. jacetanus*, as presented in Table 1 and Table 2, identified a range of known alkaloids, including assoanine, oxoassoanine, lycorine, pseudolycorine, 9-*O*-methylpseudolycorine, kirkine, and cantabricine. Notably, this extract also harbors four not identified structural components. Furthermore, the alkaloids anhydrolycorine, lycorine, galanthamine, lycoraminone, lycoramine, narwedine, haemanthamine, tazettine, epimacronine, 5,6-dihydrobicolorine, ismine, galanthindole, and two unidentified structures have been quantified among the alkaloid profiling of *N. jonquilla* (Table 1 and Table 2).

In an effort to elucidate the connections between alkaloid biosynthesis and phylogenetic relationships, Berkov and co-authors [16] determined the alkaloid patterns of 22 species and 3 hybrids from 7 main sections of the genus *Narcissus*, including the species *N. confusus*, *N. genesii-lopezii*, *N. alcaracensis*, *N. yepesii*, *N. hedraeanthus*, *N. assoanus*, *N. jonquilla*, and *N. tazetta*. The authors concluded that the chemical diversity within the *Narcissus* genus suggests that alkaloid biosynthesis, similar to floral morphology, is a dynamic process marked by extensive diversification and convergent evolution. They suggested that the biosynthesis of bioactive compounds such as Gal may take place in different phylogenetic branches. Furthermore, they noted that closely related species, inhabiting confined and neighboring regions, exhibit analogous alkaloid profiles, with variations observed in the proportions of the main alkaloid types [16].

## 3. Materials and Methods

### 3.1. Plant Material

In April 2023, a collection of fifteen distinct wild species of *Narcissus* L. was carried out in the Iberian *Narcissus* Collection of the Torretes Biological Research Station—Botanical Garden of the UA, in Ibi (Alicante), Spain, during their flowering season. The species were authenticated by a botanist, as previously and extensively described in [12]. The fifteen species evaluated in this work were as follows: *N. assoanus* Dufour ex Schult. and Schult. f.; *N. jacetanus* Fern. Casas; *N. vasconicus* (Fern. Casas) Fern. Casas; *N. minor* L.; *N. confusus* Pugsley; *N. asturiensis* (Jord.) Pugsley; *N. hedraeanthus* (Webb and Heldr.) Colmeiro; *N. alcaracensis* S. Ríos, D. Rivera, Alcaraz and Obón; *N. bujei* (Fern. Casas) Fern. Casas; *N. pallidulus* Graells; *N. tazetta* L.; *N. jonquilla* L.; *N. genesii-lopezii* Fern. Casas; *N. yepesii* S. Ríos, D. Rivera, Alcaraz and Obón; and *N. nevadensis* Pugsley. The leaves of these fifteen different *Narcissus* L. species were collected, cut into pieces, and dried at 40 °C to preserve the stability of the alkaloids.

### 3.2. Alkaloid Extraction

The dried material from the various *Narcissus* L. species was milled into a fine powder using a rotary blade mill (Taurus, Oliana, Spain). For extraction, 1 g of the powder was macerated in methanol for three days at 25 °C. The solvent was refreshed daily with three 50 mL aliquots, and an ultrasonic bath was applied for 20 min, four times a day, to enhance the extraction. The mixture was then strained, and the solvent was evaporated under reduced pressure to obtain crude extracts. The crude extracts were acidified to pH 2 with 30 mL of 2% (*v*/*v*) sulfuric acid, followed by a wash with ethyl acetate (3 × 50 mL) to remove neutral materials. The pH of the remaining aqueous phase was subsequently adjusted to 9–10 using a 25% (*v*/*v*) ammonium hydroxide solution. Finally, alkaloids were extracted with ethyl acetate (3 × 50 mL), and the solvent was evaporated to yield the dried alkaloid extract (AE).

### 3.3. Alkaloid Analysis

A total of 2 mg of each sample were dissolved in 1 mL of methanol containing 25 µg·mL^−1^ of codeine, used as the internal standard, and injected into a gas chromatograph (Agilent Technologies 6890N, Agilent Technologies, Santa Clara, CA, USA) coupled with mass spectrometry (Agilent Technologies 5975, Agilent Technologies, Santa Clara, CA, USA), both of which were obtained from Hewlett Packard, Palo Alto, CA, USA. The equipment was operated using electronic impact ionization (EI) at 70 eV with an autoinjector, 7683B Series (Agilent Technologies, Santa Clara, CA, USA). The column was a Tecknokroma TR- 45232 Sapiens-X5MS (30 m × 0.25 mm, film thickness 0.25 μm), and the splitless injection volume was 1 μL. The temperature gradient was as follows: 12 min at 100 °C, 100–180 °C at 15 °C/min, 1 min of retention at 180 °C, 180–300 °C at 5 °C/min, and 10 min of holding at 300 °C. The injector and detector temperatures were 250 and 280 °C, respectively. The carrier gas (He) flow rate was 1 mL·min^−1^.

### 3.4. Alkaloid Identification

The identification of alkaloids present in each *Narcissus* L. sample involved the comparison of the electron ionization (EI) fragments and the Kovats Retention Indices (RI) obtained for each compound against a reference library of authentic AA. This library was created by the Natural Products Research Group of the Faculty of Pharmacy and Food Sciences at the University of Barcelona, Spain. The structures of these reference compounds have been definitively established using a combination of spectroscopic techniques, including nuclear magnetic resonance (NMR), ultraviolet (UV), circular dichroism (CD), and mass spectrometry (MS). The software AMDIS 2.64 (Automatic Mass Spectral Deconvolution and Identification System) was used to process the mass spectral data. Additionally, the RI values were determined by referencing the compounds to a standard n-hydrocarbon calibration mixture containing *n*-alkanes from C9 to C30.

### 3.5. Alkaloid Quantification

A relative quantification of the AA present in each *Narcissus* L. sample was performed. A galanthamine calibration curve was constructed using concentrations ranging from 10 to 80 µg·mL^−1^. Codeine, at a fixed concentration of 25 µg·mL^−1^, was used as the internal standard. Peak areas were determined manually, considering the most abundant ions in the mass spectrum (*m*/*z*) for each compound. Typically, the base peak was used, which is *m*/*z* 286 for galanthamine and *m*/*z* 299 for codeine. For each solution, the ratio between the values obtained for galanthamine and codeine (internal standard) in each solution was plotted against the corresponding concentration of galanthamine. This plot generated the calibration curve and its equation (y = 0.0632x − 0.4557; R^2^ = 0.9962). All data were normalized to the area of the internal standard (codeine) to account for variations during sample preparation or instrument response. Subsequently, the equation obtained from the galanthamine calibration curve was used to calculate the amount of each alkaloid present in the samples. While this method does not provide the absolute concentrations, it is well-suited for comparing relative amounts of specific alkaloids between different samples. Additionally, this approach allows for comparisons with previously quantified Amaryllidaceae plants analyzed using the same method [35]. The results are expressed as micrograms of galanthamine (Gal) equivalent per 100 milligrams of dry weight (DW) of the plant material (µg GAL·100 mg^−1^ DW). Data analysis was performed using Microsoft Excel 2016 software.

## 4. Conclusions

This study reported the alkaloid profile of fifteen different species of *Narcissus* L. collected in Spain. Notably, the species *N. confusus* and *N. bujei* revealed a high content of Gal, while the species *N. pallidulus* showed a very high concentration of *Sceletium*-type structures. This is the first alkaloid report for the species *N. minor* and *N. nevadensis*, and interestingly, *N. minor* also showed the presence of *Sceletium*-type compounds. The species *N. assoanus* exhibited a high concentration and structural diversity of not identified compounds, highlighting its potential as a novel source of AA.

## Figures and Tables

**Table 1:** Alkaloids identified in *Narcissus* L. species by GC-MS. Values are expressed in μg Gal·100 mg^−1^ DW.

Alkaloid	MW	RI	A	B	C	D	E	F	G	H	I	J	K	L	M	N	O
**Lycorine-type**			**61.7**	**78.5**	**44.7**	**27.2**	**-**	**-**	**-**	**90.9**	**-**	**-**	**-**	**14.3**	**-**	**100.6**	**31.0**
Anhydrolycorine	251	2505.1	-	-	3.0	-	-	-	-	3.9	-	-	-	5.7	-	-	-
Norpluviine	273	2559.7	-	-	-	-	-	-	-	33.5	-	-	-	-	-	36.1	-
Assoanine	267	2576.1	41.5	67.4	2.5	18.7	-	-	-	5.4	-	-	-	-	-	-	3.9
Kirkine	273	2595.8	-	2.5	-	4.0	-	-	-	12.8	-	-	-	-	-	-	-
11,12-Dehydroanhydrolycorine	249	2597.7	-	-	3.6	-	-	-	-	-	-	-	-	-	-	-	3.7
Lycorine	287	2719.9	-	2.8	27.0	-	-	-	-	-	-	-	-	8.6	-	-	-
9-*O*-Methylpseudolycorine	303	2743.4	-	1.9	-	-	-	-	-	-	-	-	-	-	-	-	-
Sternbergine	331	2758.4	13.8	-	-	2.4	-	-	-	-	-	-	-	-	-	-	-
Hippamine	301	2651.6	-	-	2.5	-	-	-	-	-	-	-	-	-	-	-	-
Galanthine	317	2675.3	-	-	3.0	-	-	-	-	35.3	-	-	-	-	-	64.5	23.4
Pseudolycorine	289	2781.2	6.4	1.9	-	2.1	-	-	-	-	-	-	-	-	-	-	-
2-Hydroxyanhydrolycorine	267	2800.8	-	-	3.1	-	-	-	-	-	-	-	-	-	-	-	-
Oxoassoanine	281	2905.9	-	2.0	-	-	-	-	-	-	-	-	-	-	-	-	-
**Galanthamine-type**			**-**	**-**	**5.4**	**4.0**	**324.1**	**-**	**8.0**	**-**	**113.0**	**-**	**12.2**	**75.8**	**32.9**	**-**	**-**
Galanthamine	287	2416.3	-	-	-	-	301.0	-	3.0	-	103.2	-	5.1	22.9	12.3	-	-
Lycoraminone	287	2447.8	-	-	-	-	-	-	-	-	-	-	-	16.6	-	-	-
Lycoramine	289	2424.1	-	-	2.4	-	-	-	-	-	-	-	4.0	19.5	-	-	-
Sanguinine	273	2425.6	-	-	-	-	5.4	-	-	-	-	-	3.1	-	4.3	-	-
*N*-Demethylgalanthamine	273	2446.4	-	-	-	-	12.4	-	-	-	-	-	-	-	11.7	-	-
*O*-Demethyllycoramine	275	2460.4	-	-	3.0	-	-	-	-	-	-	-	-	-	-	-	-
Narwedine	285	2477.8	-	-	-	-	5.3	-	-	-	9.8	-	-	16.8	-	-	-
Habranthine	303	2533.6	-	-	-	-	-	-	5.0	-	-	-	-	-	-	-	-
Galanthamine *N*-oxide	303	2552.4	-	-	-	4.0	-	-	-	-	-	-	-	-	-	-	-
*O*-Methylleucotamine	373	2839.2	-	-	-	-	-	-	-	-	-	-	-	-	4.6	-	-
**Haemanthamine-type**			**13.0**	**2.9**	**2.7**	**27.7**	**92.8**	**52.7**	**38.8**	**-**	**208.4**	**5.2**	**105.7**	**9.4**	**32.0**	**-**	**-**
*O*-Methylmaritidine	301	2475.8	-	-	-	-	-	-	-	-	-	-	81.0	-	-	-	-
Vittatine	271	2479.6	-	-	-	-	-	-	9.0	-	-	-	-	-	-	-	-
Maritidine	287	2514.4	-	-	-	-	-	-	19.3	-	-	-	20.9	-	-	-	-
Papyramine/epi-papyramine	317	2537.8	-	-	-	-	-	-	-	-	-	-	3.8	-	-	-	-
8-*O*-Demethylmaritidine	273	2543.4	-	-	-	-	-	-	-	-	-	-	-	-	32.0	-	-
Deacetylcantabricine	275	2570.3	-	-	-	6.0	-	-	7.2	-	-	-	-	-	-	-	-
Haemanthamine	301	2620.3	-	-	2.7	-	92.8	52.7	-	-	141.1	5.2	-	9.4	-	-	-
11-*O*-Acetylhaemanthamine	343	2667.5	-	-	-	-	-	-	-	-	67.3	-	-	-	-	-	-
Cantabricine	317	2678.7	13.0	2.9		21.7			3.3								
***Sceletium*-type**			**-**	**-**	**-**	**6.3**	**-**	**-**	**-**	**-**	**-**	**139.9**	**-**	**-**	**-**	**-**	**-**
6-Epimesembrenol	289	2342.7	-	-	-	-	-	-	-	-	-	7.7	-	-	-	-	-
Mesembrenol	289	2351.8	-	-	-	-	-	-	-	-	-	4.4	-	-	-	-	-
6-Epimesembranol	291	2378.5	-	-	-	-	-	-	-	-	-	30.4	-	-	-	-	-
Mesembrine	289	2382.1	-	-	-	-	-	-	-	-	-	21.8	-	-	-	-	-
Mesembrenone	287	2393.6	-	-	-	2.8	-	-	-	-	-	71.0	-	-	-	-	-
2-Oxomesembrenone	301	2650.4	-	-	-	3.5	-	-	-	-	-	-	-	-	-	-	-
7,7a-Dehydromesembrenone	285	2688.2	-	-	-	-	-	-	-	-	-	4.6	-	-	-	-	-
**Pretazettine-type**			**-**	**-**	**2.7**	**-**	**53.2**	**58.7**	**25.5**	**-**	**46.5**	**4.0**	**4.4**	**32.9**	**-**	**-**	**-**
*O*-Methyltazettine	345	2614.5	-	-	-	-	3.8	14.7	-	-	-	-	-	-	-	-	-
Tazettine	331	2631.6	-	-	2.7	-	46.8	38.7	15.9	-	38.9	4.0	4.4	27.0	-	-	-
3-*O*-Methyltazettine	317	2691.6	-	-	-	-	-	-	3.4	-	-	-	-	-	-	-	-
Epimacronine	329	2775.2	-	-	-	-	2.6	5.3	6.2	-	7.6	-	-	5.9	-	-	-
**Homolycorine-type**			**-**	**-**	**2.9**	**1.9**	**21.7**	**-**	**-**	**62.9**	**135.8**	**5.4**	**49.0**	**-**	**71.1**	**135.1**	**47.2**
*O*-Methyllycorenine	331	2490.4	-	-	-	-	-	-	-	17.9	-	-	-	-	-	-	8.4
Homolycorine	315	2768.5	-	-	2.9	1.9	21.7	-	-	45.0	135.8	5.4	27.8	-	71.1	135.1	34.7
8-*O*-Demethylhomolycorine	301	2727.5	-	-	-	-	-	-	-	-	-	-	21.2	-	-	-	4.1
**Narciclasine-type**			**-**	**-**	**-**	**-**	**2.7**	**8.9**	**6.7**	**-**	**8.9**	**-**	**-**	**7.6**	**-**	**-**	**-**
Trisphaeridine	223	2299.9	-	-	-	-	-	4.4	3.1	-	-	-	-	-	-	-	-
5,6-Dihydrobicolorine	239	2342.6	-	-	-	-	2.7	4.5	3.6	-	8.9	-	-	7.6	-	-	-
**Ismine-type**			**-**	**-**	**2.5**	**-**	**14.9**	**15.9**	**6.2**	**-**	**10.6**	**-**	**-**	**7.2**	**-**	**-**	**-**
Ismine	257	2283.7	-	-	2.5	-	14.9	15.9	6.2	-	10.6	-	-	7.2	-	-	-
**Galanthindole-type**			**-**	**-**	**2.3**	**-**	**31.6**	**37.6**	**10.4**	**-**	**-**	**-**	**-**	**9.4**	**-**	**-**	**-**
Galanthindole	281	2498.7	-	-	2.3	-	31.6	37.6	10.4	-	-	-	-	9.4	-	-	-
**Montanine-type**			**-**	**-**	**-**	**-**	**-**	**-**	**-**	**-**	**-**	**-**	**-**	**-**	**-**	**25.1**	**-**
Pancratinine C	287	2579.2	-	-	-	-	-	-	-	-	-	-	-	-	-	25.1	-
**Total Alkaloids Identified**			**74.7**	**81.4**	**63.2**	**67.1**	**541.0**	**173.8**	**95.6**	**153.8**	**523.2**	**154.5**	**171.3**	**156.6**	**136.0**	**260.8**	**78.2**

A = *N. assoanus* Dufour ex Schult. and Schult.f.; B = *N. jacetanus* Fern. Casas; C = *N. vasconicus* (Fern.Casas) Fern.Casas; D = *N. minor* L.; E = *N. confusus* Pugsley; F = *N. asturiensis* (Jord.) Pugsley; G = *N. hedraeanthus* (Webb and Heldr.) Colmeiro; H = *N. alcaracensis* S.Ríos, D.Rivera, Alcaraz and Obón; I = *N. bujei* (Fern.Casas) Fern.Casas; J = *N. pallidulus* Graells; K = *N. tazetta* L.; L = *N. jonquilla* L.; M = *N. genesii-lopezii* Fern.Casas; N = *N. yepesii* S.Ríos, D.Rivera, Alcaraz and Obón; O = *N. nevadensis* Pugsley; MW = molecular weight; RI = retention index.

**Table 2:** Not identified alkaloids quantified in *Narcissus* L. species by GC-MS. Values are in μg Gal·100 mg^−1^ DW.

Not Identified (NI)	MW	RI	A	B	C	D	E	F	G	H	I	J	K	L	M	N	O
NI-1	269	2239.0	-	-	-	-	-	-	3.3	-	-	-	-	-	-	-	-
NI-2	283	2329.0	-	-	-	-	-	-	4.0	-	-	-	-	-	-	-	-
NI-3	269	2350.1	-	-	-	-	-	-	3.4	-	-	-	-	-	-	-	-
NI-4	257	2391.8	-	-	-	-	-	-	-	-	-	-	4.1	-	-	-	-
NI-5	303	2458.1	-	-	-	-	-	-	-	-	-	6.9	-	-	-	-	-
NI-6	299	2462.3	-	-	-	-	-	6.6	-	-	-	-	-	7.1	-	-	-
NI-7	305	2469.3	-	-	-	-	-	-	-	-	-	5.3	-	-	-	-	-
NI-8	329	2523.8	-	23.5	-	4.3	-	-	-	-	-	-	-	-	-	-	-
NI-9	317	2391.8	-	-	-	-	-	-	-	-	-	-	4.8	-	-	-	-
NI-10	301	2490.4	-	-	-	-	-	-	-	5.5	-	-	-	-	-	-	-
NI-11	303	2548.2	-	-	-	-	-	-	-	-	-	3.9	-	-	-	-	-
NI-12	287	2560.5	-	12.4	-	-	-	-	-	-	-	-	-	-	-	-	-
NI-13	289	2572.4	-	-	-	-	-	-	-	-	-	4.3	-	-	-	-	-
NI-14	315	2592.8	14.0	-	-	-	-	-	-	-	-	-	-	-	-	-	-
NI-15	345	2615.6	-	-	-	-	-	9.4	-	-	-	-	-	-	-	-	-
NI-16	251	2688.4	8.3	-	-	-	-	-	-	-	-	-	-	-	-	-	-
NI-17	355	2699.2	24.4	-	-	-	-	-	-	-	-	-	-	-	-	-	-
NI-18	345	2709.3	4.4	-	-	-	-	-	-	-	-	-	-	-	-	-	-
NI-19	373	2729.3	9.6	-	-	-	-	-	-	-	-	-	-	-	-	-	-
NI-20	315	2741.1	-	2.0	-	-	-	-	-	-	-	-	-	-	-	-	-
NI-21	343	2752.6	-	-	-	-	-	-	-	-	-	-	-	5.9	-	-	-
NI-22	359	2766.0	-	-	-	2.0	-	-	-	-	-	-	-	-	-	-	-
NI-23	279	2766.7	-	2.1	-	-	-	-	-	-	-	-	-	-	-	-	-
NI-24	297	2772.1	-	-	2.4	-	-	-	-	-	-	-	-	-	-	-	5.3
NI-25	315	2774.0	-	-	-	-	61.8	-	-	45.0	-	-	-	-	24.7	-	-
NI-26	329	2782.3	-	-	-	-	-	-	3.0	-	-	-	-	-	-	-	-
NI-27	355	2800.9	-	-	-	-	-	-	4.2	-	-	-	-	-	-	-	-
NI-28	313	2778.6	-	-	-	-	-	-	-	-	-	-	-	-	-	-	3.7
NI-29	345	2810.8	4.2	-	-	-	-	-	-	-	-	-	-	-	-	-	-
NI-30	331	2858.1	11.5	-	-	4.1	-	-	-	-	-	-	-	-	-	-	-
**Total of not identified**			**76.4**	**40.0**	**2.4**	**10.4**	**61.8**	**16.0**	**17.9**	**50.5**	**-**	**20.4**	**8.9**	**13.0**	**24.7**	**-**	**9.0**

A = N. assoanus; B = N. jacetanus; C = N. vasconicus; D = N. minor; E = N. confusus; F = N. asturiensis; G = N. hedraeanthus; H = N. alcaracensis; I = N. bujei; J = N. pallidulus; K = N. tazetta; L = N. jonquilla; M = N. genesii-lopezii; N = N. yepesii; O = N. nevadensis; MW = molecular weight; RI = retention index.

**Table 3:** Literature information about alkaloid-type scaffolds previously identified in fifteen different species of *Narcissus* L.

Code	Species	Alkaloid-Type Scaffold	Reference
A	*N. assoanus*	Lycorine-type	[20,21]
B	*N. jacetanus*	Lycorine-type	[22]
C	*N. vasconicus*	Homolycorine-, and lycorine-type	[23]
D	*N. minor*	-	-
E	*N. confusus*	Galanthamine-, haemanthamine-, homolycorine-, ismine-, lycorine-, narciclasine-, and pretazettine-type	[13,16,24,25,26]
F	*N. asturiensis*	Ismine-, haemanthamine-, and pretazettine-type	[27]
G	*N. hedraeanthus*	Galanthamine, haemanthamine, lycorine-, narciclasine-, and pretazettine-type	[16]
H	*N. alcaracensis*	Haemanthamine-, homolycorine-, and lycorine-type	[16]
I	*N. bujei*	Bujeine-, haemanthamine-, and homolycorine-type	[28]
J	*N. pallidulus*	Galanthamine-, haemanthamine-, homolycorine-, lycorine-, pretazettine-, and *Sceletium*-type	[15,29]
K	*N. tazetta*	Galanthamine-, haemanthamine-, homolycorine-, lycorine-, montanine-, norbelladine-, and pretazettine-type	[30,31,32]
L	*N. jonquilla*	Galanthamine-, haemanthamine-, lycorine-, narciclasine-, and pretazettine-type	[16,33,34]
M	*N. genesii-lopezii*	Galanthamine-, haemanthamine-, homolycorine-, and lycorine-type	[16]
N	*N. yepesii*	Galanthamine-, haemanthamine-, homolycorine-, lycorine-, and montanine-type	[16]
O	*N. nevadensis*	-	-

## Data Availability

Data are contained within the article.
